# Impact of Cognitive Reserve and Premorbid IQ on Cognitive and Functional Status in Older Outpatients

**DOI:** 10.3390/brainsci11070824

**Published:** 2021-06-22

**Authors:** Maria C. Quattropani, Alberto Sardella, Francesca Morgante, Lucia Ricciardi, Angela Alibrandi, Vittorio Lenzo, Antonino Catalano, Giovanni Squadrito, Giorgio Basile

**Affiliations:** 1 Department of Clinical and Experimental Medicine, University of Messina, 98125 Messina, Italy; maria.quattropani@unime.it (M.C.Q.); fmorgante@gmail.com (F.M.); catalano.antonino@unime.it (A.C.); giovanni.squadrito@unime.it (G.S.); giorgio.basile@unime.it (G.B.); 2 Neurosciences Research Centre, Molecular and Clinical Sciences Research Institute, St George’s University of London, London SW17 0RE, UK; lucia.ricciardi2@gmail.com; 3 Medical Research Council Brain Network Dynamics Unit, Nuffield Department of Clinical Neurosciences, Oxford OX1 3TH, UK; 4 Unit of Statistical and Mathematical Science, Department of Economics, University of Messina, 98123 Messina, Italy; angela.alibrandi@unime.it; 5 Department of Social and Educational Sciences of the Mediterranean Area, “Dante Alighieri” University for Foreigners of Reggio Calabria, 89125 Reggio Calabria, Italy; v.lenzo@unidarc.it

**Keywords:** cognitive reserve, premorbid intelligence, cognitive functioning, functional status, frailty, gait speed, handgrip strength, older adults, clinical psychology

## Abstract

The study aimed to investigate cross-sectionally the associations of cognitive reserve (CR) and premorbid IQ with cognitive and functional status in a cohort of older outpatients. Additionally, we evaluated the association of CR and premorbid IQ with the worsening of patients’ cognitive status at one-year follow-up. We originally included 141 outpatients (mean age 80.31 years); a telephone-based cognitive follow-up was carried out after one year, including 104 subjects (mean age 80.26 years). CR (β = 0.418), premorbid IQ (β = 0.271) and handgrip strength (β = 0.287) were significantly associated with the MMSE score. The cognitive worsening at follow-up was associated with lower CR, lower MMSE score, reduced gait speed and frailty exhibited at baseline. Univariate linear regressions showed that CR was associated with handgrip strength (β = 0.346), gait speed (β = 0.185), autonomy in basic (β = 0.221) and instrumental (β = 0.272) daily activities, and frailty (β = −0.290); premorbid IQ was significantly associated with autonomy in instrumental daily activities (β = 0.211). These findings highlight the need for integrating CR and premorbid IQ with physical and motor measures when appraising predictors of cognitive decline in the elderly population. The study also newly extends the link of CR and premorbid IQ to the functional status in older adults.

## 1. Introduction

The contribution of multidimensional antecedent factors involved in aging trajectories has been largely debated, given the bio-psycho-social nature of different age-related medical conditions, as well as of cognitive processes [1,2,3,4]. In this context, the concept of reserve has been originally suggested as a core of protective factors against the onset of negative age-related outcomes [5]. Along with passive reserves, such as the brain reserve, each individual can arrange a different variety of factors defined as active, in that they can be actively implemented and improved throughout the life course. Within the range of active reserves, the construct of cognitive reserve (CR) has been developed to describe individual differences in vulnerability to cognitive and functional decline along aging. Precisely, the CR hypothesis better explains the observed discrepancies between the amount of brain neuropathology and the degree of cognitive or functional impairment among individuals [6].

CR has always represented a wide construct, encompassing different aspects. According to the model originally proposed by Stern, education level, occupational attainment and engagement in activities that are cognitively, socially, and physically stimulating have been selected as the most representative factors of CR [7,8]. An established consensus has been reached in suggesting that such factors contributing to CR are associated with a later onset and/or a decreased risk of dementia [9]. Indeed, the purpose of previous studies has been to explore their direct or indirect contribution mainly on cognitive trajectories towards dementia [10,11].

In line with the Stern model, premorbid intelligence quotient (IQ) has been considered a further variable contributing to enhance individual CR [7]. Previous studies have highlighted the positive effect of childhood IQ on individual cognitive evolution during adulthood and old age [12], with an increased risk of developing dementia in women with lower IQ, compared to men [13]. Vocabulary or reading tests are generally considered reliable tools to measure premorbid IQ, since these functions appear to remain preserved along aging, even in presence of neurodegenerative or vascular pathologies [14].

The majority of the studies that investigated CR in the last decades has often measured CR in terms of single proxies, with education level as the most frequently accounted factor [15,16]. That is, the necessity to achieve an integrated measure of CR has been emphasized. Recently, a growing number of studies have operationalized the construct of CR, by employing accurate tools that summarize the integrated contribution of the various CR indices [17,18,19,20].

Besides the decline of cognitive performances, older adults may often exhibit a worsening of their functional status as witnessed by reduced physical and motor performances [21,22]. This physical and motor progressive deterioration exposes older adults to higher risk of negative functional outcomes, such as frailty, which denotes a condition characterized by reduced homeostatic reserves and increased individual vulnerability to stressors, and increases the risk of negative consequences as disability and mortality [23]. Frailty could be described in terms of deficit accumulation; accordingly, frailty status results from the amount of health deficits an individual has accumulated during the life course [24]. The strong relationship between motor and cognitive performances in the elderly population is supported by several studies showing that the development of cognitive impairment among older adults is associated to a reduction in walking speed or in muscle strength [25,26]. Similarly, deficit accumulation-based frailty has been recently associated with the evolution of cognitive trajectories among older adults [27,28].

How CR can be associated with such physical and motor indices and with frailty is a current topic of scientific debate. For instance, it has been previously suggested that CR may modulate the association of daily autonomy and motor performances with cognitive status in older subjects [29], or may positively influence the physical rehabilitation of patients with peculiar neurodegenerative diseases [30]. Similarly, the beneficial role of single CR proxies, especially education and occupation, has been highlighted in previous studies exploring frailty among older adults [16].

Based on these premises, we investigated the association of CR and premorbid IQ with cognitive and functional status in a cohort of older adults. In line with the Stern model, we decided to evaluate separately CR and premorbid IQ, considering the latter an additional factor able to enhance CR.

We first designed a cross-sectional study to investigate the association of CR, premorbid IQ, physical and motor measures with cognitive status. This represents one of the novelties of the study, since we adopted a multidimensional approach that integrated the assessment of CR, premorbid IQ, physical and motor performances. These are factors commonly associated with cognitive status, but have not been jointly assessed in elderly subjects yet.

Furthermore, we aimed to cross-sectionally explore the associations of CR and premorbid IQ with common indexes of functional status (i.e., handgrip strength, gait speed, daily autonomy and frailty), which denotes an additional novelty of the study.

Ultimately, we evaluated the potential association of baseline measures of CR, premorbid IQ and functional status with the worsening of cognitive status at one-year follow-up.

## 2. Materials and Methods

### 2.1. Subject and Study Design

This was an observational cross-sectional study, with a follow-up assessment of cognitive status, involving a cohort of patients attending the Geriatrics and Multidimensional Evaluation Outpatients Clinic of the University Hospital “Gaetano Martino”, Messina, Italy.

All consecutive patients with an age ≥ 65 years attending the outpatient geriatric clinic for their routine appointment between April and May 2019 were invited to participate in the study. We excluded subjects with a major neurocognitive disorder according to the DSM-5 diagnostic criteria [31], which would limit the comprehensive administration of the scales and the execution of the tasks. We also excluded patients with severe functional and sensory limitations (e.g., subjects in wheelchairs and/or not able to walk; subjects with severe limitations in the upper limbs; subjects with severe visual and/or hearing impairments). The severity of the sensory deficits (visual and hearing) was either evidenced by previous diagnoses reported by the patients, or clinically judged by the same geriatrician during the visit; we excluded those patients who, due to these limitations, were not able to adequately, and above all entirely, sustain the evaluation. The exclusion of patients with severe physical limitations was also justified by the fact that the assessment of the frailty status was based on measurements of physical performances, besides other factors.

Patients included in the study underwent a baseline multidimensional evaluation, which consisted of the assessment of cognitive and functional status. A follow-up evaluation of the participants’ cognitive status was carried out between April and May 2020, by telephone interviews due to COVID-19 pandemic restrictions. In order to avoid potential rater-related bias, one trained clinical psychologist conducted the phone calls and administered the test.

### 2.2. Outcome Measures

#### 2.2.1. Cognitive Functioning

The MMSE was administered at baseline to screen for global cognitive functioning [32].

For the follow-up assessment, we employed the validated Italian Telephone version of the MMSE (Itel-MMSE). High interrater and test-retest reliabilities were reported (0.82 ± 0.90 and 0.90 ± 0.95, respectively); furthermore, the total scores of the MMSE and Itel-MMSE versions were found strongly correlated (r = 0.85) [33].

Compared to the original MMSE, the Itel-MMSE includes each temporal/spatial orientation item, except for “*On which floor are we?*”. The original items concerning memory, attention and calculation are fully reported even in the Itel-MMSE. The principal difference from the original MMSE concerns the section dedicated to the evaluation of language and praxia. Accordingly, in the Itel-MMSE the patient is asked to name “*the thing currently used to talk*” to the examiner (i.e., telephone); the items dedicated to the execution of motor sequences, the execution of a written command (i.e., “*Close your eyes*”), the sentence writing and the copy of pentagons are not included in the Itel-MMSE. The test administration usually lasts about five minutes; the total score ranges from 0 to 22, with higher scores expressive of better cognitive status. For the purpose of our study, we estimated the expected MMSE score based on the score obtained on the Itel- MMSE, by the use of an algorithm, as suggested by the standard procedure of the test [33].

#### 2.2.2. Functional Status

The functional status was evaluated in terms of physical and motor performances, daily autonomy, and frailty status. Motor and physical performances were measured by means of the 4 m gait speed (expressed as meter per second) and handgrip strength (expressed in kilograms, measured by a Jamar dynamometer), respectively.

Daily life autonomy was evaluated at baseline through the Basic Activities of Daily Life (BADL) scale [34] and the Instrumental Activities of Daily Life (IADL) scale [35]. Higher scores reflect a better autonomy in performing daily activities.

Frailty status was evaluated by the calculation of the 35-deficit Frailty Index (FI), according to the standard procedure [36]. The FI is usually expressed as the ratio of health-related deficits present to the total number of deficits considered; consistently, higher FI scores correspond to a more severe frailty status. According to this deficit accumulation model, subjects with a FI ≥ 0.25 are commonly categorized as frail [24]. The thirty-five variables that were evaluated for the calculation of the FI are provided as Supplementary Material (Table S1).

#### 2.2.3. Cognitive Reserve

We administered the Cognitive Reserve Index questionnaire (CRIq) to explore participants’ cognitive reserve [37], in line with the theoretical model proposed by Stern [7]. The questionnaire was administered by the same clinical psychologist to the patients. The CRIq includes some preliminary demographic data (date and place of birth, gender, nationality, marital status), followed by 20 items assembled into three sections, investigating the subject’s educational status, occupational level and leisure time activities. Precisely, the education section investigates years of education, plus the achievement of any other type of additional training. The occupational level section investigates five different levels of work activities (i.e., unskilled or manual work; skilled manual work; skilled nonmanual or technical work; professional occupation; highly intellectual occupation); work activity is collected as the number of years in each profession over the lifespan. Ultimately, the leisure time section investigates intellectual, social and physical activities carried out during leisure time; these data are collected in terms of frequency, as well as the number of years each activity had been carried out by the subject [37].

The questionnaire returns subscores for each domain, together with a total score; the latter was used in the current study. Higher scores denote a greater cognitive reserve.

#### 2.2.4. Premorbid IQ

In general, the evaluation of IQ is broadly carried out when information on individual intellectual performances are needed. Instead, the evaluation of premorbid IQ allows researchers to estimate how a neurological impairment has affected patients’ intellectual capacity. Premorbid IQ is usually measured by reading-based tasks, based on the assumption that reading ability is preserved across time contrary to the functioning of other cognitive processes, which deteriorates with age.

For the purpose of our study, the Brief Intelligence Test (originally named *Test di Intelligenza Breve*—TIB) was administered to estimate the premorbid IQ [38], as an additional factor contributing to cognitive reserve [7]. The Italian TIB was developed from the National Adult Reading Test (NART) [39]. Consistently, the TIB is based on reading thirty-four words of rare or uncommon use, with an irregular accent, together with additional twenty control-words of frequent use [40]. The test was administered by a clinical psychologist; the subject was asked to read each word aloud. The TIB returns a total score of premorbid IQ, calculated by a predefined algorithm [33]; higher scores correspond to higher premorbid IQ [40].

### 2.3. Data Analysis

We performed a post hoc power analysis, using the G*Power software (version 3.1.9.6), which reported a statistical power >90, with a critical F = 3.06 (considering the α error probability of 0.05 and the total sample size of 141).

According to the skewness and kurtosis values, the variables resulted normally distributed. Descriptive data were reported in terms of mean, standard deviation (SD) and/or percentage. The correlations between variables were explored by Pearson’s correlation coefficient (data reported as Supplementary Material, Table S2).

To test the associations of the investigated variables with cognitive status (dependent variable), we performed multivariate linear regressions that were used for both cross-sectional and longitudinal analysis. Precisely, we performed hierarchical three-step multivariate linear regressions, including sociodemographic variables in the first step (i.e., age, gender, and years of education), motor and physical variables in the second step (i.e., handgrip strength and gait speed), and CRIq and the TIB in the third step.

Univariate linear regressions were executed to explore the linear associations between the CRIq and the TIB with functional indexes (i.e., handgrip strength, gait speed, daily autonomy and FI).

Differences between baseline and follow-up cognitive scores were calculated with a paired-sample Student’s t-test. The variation between the MMSE score obtained at follow-up and that obtained baseline was calculated, and expressed as the mean Δ (t1 − t0) ± SD. Subjects were consequently classified in two groups, based on the occurrence or not of a “worsening” in their cognitive performances.

Data were analyzed using the IBM SPSS 22 statistical software. Values of *p* < 0.05 were considered statistically significant.

## 3. Results

### 3.1. Demographic and Clinical Features of the Study Sample

The original sample consisted of 141 outpatients (42 males and 99 females), with a mean age of 80.31 ± 6.84 years. Each patient was native Italian. Mean MMSE score was 22.6 ± 4.5; participants were partially independent in their basic and instrumental daily activities (mean BADL score = 4.23 ± 1.5; mean IADL score = 3.67 ± 1.4, respectively). The sample showed a mean FI score of 0.25 ± 0.11, with FI scores ranging from 0.05 to 0.50. The baseline main sociodemographic and clinical characteristics of the sample are summarized in Table 1.

From the original sample, we were able to contact by telephone 115 patients. Eleven of them declined to undergo the evaluation and 104 patients available for follow-up evaluation (mean age = 80.26 ± 6.39; 33 males, 71 females). As per the FI calculated at baseline, the follow-up sample consisted of 50 subjects classified as frail and 54 subjects classified as not frail. The follow-up cognitive evaluation was carried out 12 months after the baseline evaluation. The variation (Δ) between the MMSE score obtained at follow-up and baseline was of −0.033 ± 0.07 (−3.3%). The Δ of the two MMSE was positively correlated with baseline MMSE (r = 0.492; *p* < 0.001), gait speed (r = 0.224; *p* = 0.024), CRIq (r = 0.333; *p* = 0.001) and TIB (r = 0.229; *p* = 0.019), and it was negatively correlated with the FI (r = −0.333; *p* = 0.001).

Cognitive functioning at follow-up was worse (mean MMSE score = 22.001 ± 4.93) compared to baseline (mean MMSE score = 22.81 ± 4.3), and the difference was statistically significant (t = 8.055; *p* < 0.001).

**Figure 1:**
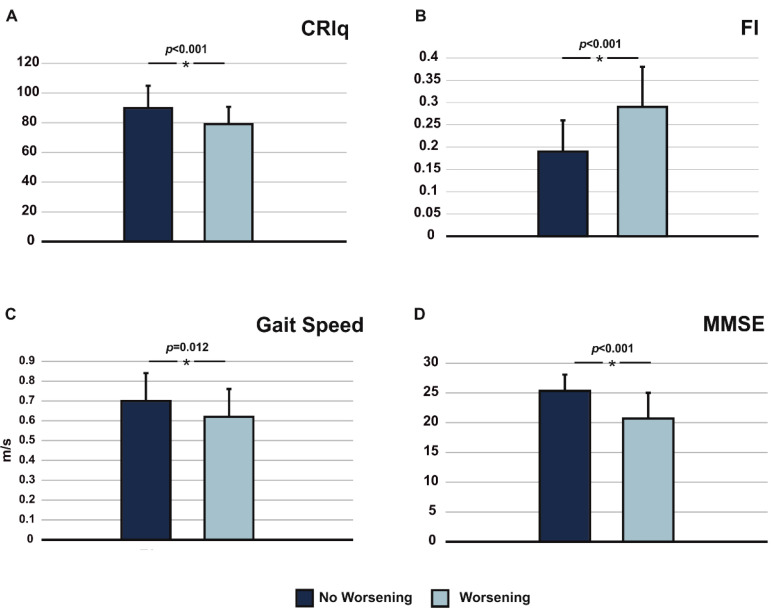
Comparisons of subjects with or without a cognitive worsening at one-year follow-up, based on baseline CRIq score (panel **A**), Frailty Index (FI) (panel **B**), gait speed (panel **C**), and MMSE score (panel **D**).

Based on the variations in the MMSE scores, a worsening of the performance was found in fifty-eight patients (“worsening group”); conversely, in the remaining forty-six patients (“not worsening group”), scores remained stable (*n* = 40) or even improved (*n* = 6). Patients with cognitive worsening at follow-up were those who exhibited significantly lower CRIq scores (mean 79.14 ± 15.02 vs. 89.87 ± 11.6) lower MMSE scores (mean 20.7 ± 4.31 vs. 25.3 ± 2.74), reduced gait speed (mean 0.62 ± 0.14 vs. 0.70 ± 0.16), and higher FI (mean 0.29 ± 0.09 vs. 0.19 ± 0.07) at baseline (Figure 1).

### 3.2. Associations of CR and Premorbid IQ with Functional Status

Univariate linear regressions revealed significant associations of the CRIq with handgrip strength (β = 0.346), and gait speed (β = 0.185), indicating that subjects with higher CR exhibited greater muscle strength and better motor performances. In addition, the CRIq was significantly associated with BADL (β = 0.221), IADL (β = 0.272) and FI (β = −0.290), indicating that subjects with higher CR appeared more autonomous in daily activities, and showed a lower degree of frailty.

The TIB was significantly associated with IADL (β = 0.211), which indicates that subjects with higher premorbid IQ were more autonomous in performing instrumental daily activities; the TIB resulted only marginally associated with the FI (β = −0.161; *p* = 0.057).

Data are reported in Table 2.

### 3.3. Association of CR and Premorbid IQ with Cognitive Status

We developed a first multivariate linear regression model with the baseline MMSE score as the dependent variable, in which we explored the association of CRIq and TIB, in the presence of additional factors that commonly influence cognitive status. In line with this purpose, we developed a hierarchical three-step multivariate linear regression model, including sociodemographic variables in the first step (i.e., age, gender, and years of education), motor and physical variables in the second step (i.e., handgrip strength and gait speed), and CRIq and the TIB in the third step. The final step (R^2^ = 0.307; *p* < 0.001) reported that handgrip strength (β = 0.287; *p* = 0.004), CRIq (β = 0.418; *p* = 0.001) and TIB (β = 0.271; *p* = 0.002) were significantly associated with the MMSE score. The multivariate linear regression model is extensively summarized in Table 3.

This evidence was confirmed also in a further multivariate linear regression, which considered the MMSE score obtained at follow-up as the dependent variable. Indeed, the final step (R^2^ = 0.273; *p* < 0.001) confirmed that CRIq (β = 0.381; *p* = 0.013), TIB (β = 0.222; *p* = 0.03), and handgrip strength (β = 0.315; *p*= 0.008) significantly contributed to the cognitive status at follow-up.

## 4. Discussion

The present study investigated the association of CR and premorbid IQ with the cognitive status of elderly individuals, taking into account the role of additional factors such as physical and motor performance. We showed that measures of CR and premorbid IQ (CRIq and TIB, respectively) and a measure of physical performance (handgrip strength) were associated with cognitive status, measured by MMSE. In addition, baseline variables associated with a worsening of cognitive performances over the one-year follow-up included a lower CR, a higher frailty score, a slower gait and a lower MMSE score.

### 4.1. Cognitive Reserve, Premorbid IQ, Functional Variables and Cognitive Performance: Need for Multidimensional Assessment

It is an established concept that subjects with a higher CR are associated with a later onset and/or a decreased risk of dementia [9]. Similarly, some evidence supports the positive contribution of premorbid IQ on cognitive trajectories [13,41]. Conversely, the association of CR and IQ with age-related functional status, though it denotes a topic of growing interest, has been little investigated to date.

Overall, our results highlight the need to incorporate the evaluation of CR, premorbid IQ, functional indexes and cognitive functioning.

The statistical models employed in our study showed that CR, premorbid IQ and handgrip strength were significantly associated with the patients’ cognitive status. Based on these results, we can suggest an integrated model in which elderly people with higher CR, higher premorbid IQ, and greater muscle strength can exhibit a better cognitive status. These findings also allow us to newly interpret the association of CR and premorbid IQ with cognitive functioning in the context of a joint cognitive-motor perspective of aging [42].

In addition, we explored the novel association of CR and premorbid IQ with measures of patients’ functional status, namely handgrip strength, gait speed, daily autonomy and frailty. Accordingly, we showed that older adults with higher CRIq have a greater muscle strength, a quicker gait speed, a better overall daily autonomy (BADL and IADL), and were less frail (as per the FI). In addition, higher premorbid IQ (TIB) was associated with a better autonomy in managing instrumental activities (IADL); moreover, frailty was also weakly associated with premorbid IQ (*p* = 0.057), an association deserving to be explored carefully in future studies.

Among the few available evidence accounting for physical performances, the CRIq has been peculiarly associated with extrapyramidal symptoms, such as tremor, bradykinesia, rigidity, postural instability and balance, in samples of patients with Parkinson’s disease [30,43]. In support of the link between CR and gait speed, the most established proxy of CR, namely education, has been recently confirmed as a potential risk factor for the Motoric Cognitive Risk (MCR) syndrome, which is a predementia syndrome combining slow gait speed and cognitive complaints [44].

The association between CR and autonomy in managing daily activities has emerged only in few studies, in which education or vocabulary abilities modulated the association between daily autonomy and gait speed with cognitive status [29,45]. Similarly, a reading task-based IQ has been recently discussed as a moderator in the relationship between daily autonomy and cognitive performances [46], in line with the established assumption that the performance of instrumental daily activities is strongly linked to the efficiency of cognitive processes [47,48].

The association of CR and premorbid IQ with frailty is a recent topic of investigation, which deserves some considerations. Frailty denotes a crucial outcome in aging studies, and it has been broadly investigated in community populations and hospital settings [49,50]. Since frailty is considered a multidimensional and dynamic condition, the hypothesis that CR and premorbid IQ might have an impact not only on cognitive trajectories but also on frailty has been increasingly debated during the last years. To date, only a limited numbers of studies tested the association of premorbid IQ with frailty [16]. Moreover, when the effect of CR on frailty was previously explored, the main methodological weakness has been the use of CR proxies such as education, professional employment or leisure activities. For instance, findings from a recent large prospective study highlighted that physically frail individuals showed lower education, lower socioeconomic status, and reduced engagement in social activities compared with robust individuals, which indirectly suggests that physically frail individuals had a lower cognitive reserve [51]. In this context, the use of a measure of CR (i.e., the CRIq) in our study represents a step forward to overcome such limitation. Another novel point of our study was to have investigated the association between premorbid IQ and frailty in an Italian population for the first time.

How should we frame the association of CR and premorbid IQ with frailty, based on our data of a similar association with cognitive performance in the elderly population? We could hypothesize that lower CR and lower premorbid IQ might have a negative impact on individual aging trajectories, which are characterized by cognitive and motor/physical decline, eventually leading to frailty [42]. The findings from the present study could suggest that CR and premorbid IQ might have a positive role along the cognitive trajectory leading to frailty. Moreover, consistently with the above-mentioned joint cognitive-motor approach to aging, our findings, though preliminary, might be also interpreted in line with the concept of cognitive frailty, which refers to a clinical condition characterized by the presence of both physical and cognitive impairment, in the absence of a manifest diagnosed dementia [52]. However, future studies are needed to better verify this hypothesis.

The accurate and operationalized evaluation of CR and premorbid IQ in older patients might also lead physicians to enrich the Comprehensive Geriatric Assessment (CGA), by accounting for the potential impact of these antecedent factors beyond cognitive trajectories [53]. In line with this integrated perspective, CR and premorbid IQ could be suggested as two antecedents that might predict both cognition and functional trajectories among older adults [54].

### 4.2. Impact of Functional Variables and CR on Worsening of Cognitive Functions

The relevance of CR in predicting the trajectory of cognitive performance over time is further confirmed by the follow-up analysis conducted in 104 outpatients, through the administration of the Itel-MMSE, the telephone based validated version of MMSE. This analysis needs to be interpreted with caution for two main reasons: (1) the use of MMSE as a measure of cognitive performance, which is not sensitive to subtle changes; (2) the relatively limited follow-up time (one year). Yet, some interesting insights were provided by this analysis.

Group analysis showed that subjects who had a worsening of cognitive performances had a lower CR (expressed by the CRIq), a higher frailty score, a slower gait and a lower baseline MMSE score. These data support the hypothesis that neurodegeneration and/or vascular brain disease may have a predilection for areas controlling gait and cognition, thus explaining the parallel deterioration of motor function and cognitive performance. Accordingly, a systematic review of 39 longitudinal studies revealed that slow gait speed is a strong predictor of incident dementia and cognitive decline in the elderly population [55]. It is conceivable to hypothesize that cognition, motor performance and frailty are interrelated domains, which might contribute to aging. Indeed, the progressive reduction of cognitive functions and the decrease of motor performances might be considered as part of a joint pathway towards age-related outcomes [42], and slow gait might be used as a reliable measure of cognitive trajectories as proposed by a recent meta-analysis [56].

### 4.3. Strengths and Limitations

One of the strengths of our study was the use of two measures of CR and premorbid IQ, namely the CRIq and the TIB, respectively. Indeed, the achievement of an operationalized assessment of CR has been a challenge over the past years, as most of the studies have often referred to a single CR proxy, such as the education level [57], or have combined different proxies through the implementation of structural equation modelling-based analyses [58]. Furthermore, according to the model proposed by Stern [7], individual IQ is considered an additional factor contributing to CR. Linguistic abilities have been broadly considered a valid index of IQ, thus, the use of a reading-based test might represent a suitable solution [7].

The CRIq has been recently suggested as a reliable instrument for the operationalized assessment of CR, which may overcome the weakness of a partial evaluation of CR and favor a better standardization of the assessment [59]. The use of CRIq has been previously promoted to explore its association with cognitive functioning in nondemented older adults [60,61], and in several clinical populations [62,63,64,65].

We also explored premorbid IQ through the reading based TIB, which may represent a quick and easy to administer tool, especially in the elderly people. This test was originally proposed to estimate premorbid IQ in subjects with cognitive impairment [40], and has been employed for the same purpose among nondemented older patients with Parkinson’s disease, together with the CRIq [66].

An additional point of strengths regards the development of a novel integrated model to explain cognitive functioning, which included CR, premorbid IQ, physical and motor performances. As novel is the joint investigation of measures of CR and premorbid IQ in relation to physical and motor performances, daily autonomy indexes and frailty.

We acknowledge that the present study has some limitations. A referral bias might have impacted on our results, as the study sample consisted of older outpatient subjects. The predominant female prevalence did not allow us to explore gender differences. Cognitive status was tested with the MMSE that is a measure of global cognition and it is used as a screening tool in clinical practice, which might not be enough sensitive to changes over a narrow period. We assigned the subjects in the “worsening” group based only on the presence of a reduction of the cognitive status at follow-up, which might have narrowed the clinical relevance of the worsening. Furthermore, the follow-up evaluation of cognitive status was carried out by telephone; although the Itel-MMSE is considered a reliable tool, the administration procedure, and the emergency context in which it was carried out, could represent further limitations. Finally, the univariate linear regressions performed to evaluate the associations of CR and premorbid IQ with functional indexes need to be interpreted as preliminary findings; future studies with more in-depth analyses are encouraged in order to narrow the risk of biased results.

## 5. Conclusions

The present study investigated the association of measures of CR and premorbid IQ with cognitive functioning in a cohort of older adults. In line with a framework integrating physical, motor and cognitive functions as a key to successful ageing, we developed a model in which CR and premorbid IQ were tested as contributors of cognitive status, in the presence of physical and motor indexes. Cognitive status was influenced by multiple variables including CR, antecedent intellectual performance (premorbid IQ) as well as physical contributors (handgrip strength). Moreover, a lower CR, a worse motor performance measured by gait speed, and a greater frailty status were associated to the worsening of cognition over time.

The present study additionally explored the associations of CR and premorbid IQ with indexes of functional status; accordingly, higher CR was associated with greater muscle strength, quicker gait speed, a better daily autonomy, and a lower frailty status; similarly, higher premorbid IQ was associated with a better autonomy in instrumental daily activities.

These data highlight the need for using operationalized measures of CR and premorbid IQ, and integrating them with physical and motor measures when appraising predictors of cognitive decline in the elderly population. The study also extends the link of CR and premorbid IQ to the functional status in older adults.

We encourage future research to clarify the link between cognitive reserve, IQ and cognitive-motor trajectories of aging, as well as their impact on sarcopenia, frailty and disability, extending these evaluations also to older populations.

Such an approach might eventually allow researchers to define intervention strategies leading to active and successful aging. The concept of active aging is central in psychology research, which highlights the importance of economic, social, and cultural engagement, besides physical. Consistent with this perspective, CR and premorbid IQ could improve subjects’ awareness of their health, and promote the adoption of prohealth behaviors and lifestyle along the life course.

## Figures and Tables

**Table 1:** Baseline sociodemographic and clinical characteristics of the sample (*n* = 141).

**Sociodemographic characteristics ^1^**	
Age (years)	80.31 ± 6.84
Gender ^2^ Male (*n*, %) Female (*n*, %)	42 (29.8)
	99 (70.2)
Education (years)	7.09 (± 3.83)
Marital status Married (*n*, %) Widow/er (*n*, %) Other (*n*, %)	71 (50.4)
	56 (39.7)
	14 (9.9)
**Clinical features ^1^**	
MMSE	22.61 (± 4.52)
Handgrip (kg)	17.25 (± 7.14)
Gait speed (m/s)	0.64 (± 0.19)
BADL ^3^	4.23 (± 1.59)
IADL ^3^	3.67 (± 2.41)
FI	0.25 (± 0.11)
Frailty status ^4^ Frail (*n*, %) Not frail (*n*, %)	71 (50.4)
	70 (49.6)
CRIq	83.33 (± 14.53)
TIB	96.34 (± 13.63)

Abbreviations: MMSE = Mini Mental State Examination; BADL = Basic Activities of Daily Life; IADL = Instrumental Activities of Daily Life; FI = Frailty Index; CRIq = Cognitive Reserve Index Questionnaire; TIB = Test di Intelligenza Breve; SD = Standard Deviation; m/s = meter per second. ^1^ Those sociodemographic and clinical variables that are not expressed as percentages are expressed as mean scores ± SD. ^2^ The variable “gender” was dichotomized as “0 = male”, “1 = female”. ^3^ BADL and IADL scores express the number of maintained functions by the subject. ^4^ Subjects with an FI ≥ 0.25 were considered frail.

**Table 2:** Associations of CR and premorbid IQ with functional variables.

	CRIq				TIB			
	B	β	t	*p*	B	β	t	*p*
Handgrip	0.170	0.346	4.35	<0.001	0.042	0.081	0.95	0.34
Gait speed	0.002	0.185	2.20	0.029	0.002	0.117	1.37	0.17
BADL	0.024	0.221	2.66	0.009	0.008	0.072	0.85	0.39
IADL	0.045	0.272	3.32	0.001	0.037	0.211	2.54	0.012
FI	−0.002	−0.290	−3.56	<0.001	−0.001	−0.161	−1.91	0.0571

Note. The CRIq and the TIB were tested as independent variables; functional measurements were considered the dependent variable of the univariate regressions. MMSE = Mini Mental State Examination; BADL = Basic Activities of Daily Life; IADL = Instrumental Activities of Daily Life; FI = Frailty Index; CRIq = Cognitive Reserve Index Questionnaire; TIB = Test di Intelligenza Breve.

**Table 3:** Hierarchical multivariate linear regression model for baseline MMSE score.

	Model Summary				Coefficients		
	Step	R^2^	F	*p*	β	t	*p*
	Step 1	0.084	4.12	0.008			
Age					−0.118	−1.43	0.15
Gender ^1^					−0.180	−2.11	0.036
Education					0.157	1.84	0.067
	Step 2	0.173	5.58	<0.001			
Age					−0.19	−0.22	0.82
Gender ^1^					0.066	0.058	0.95
Education					0.128	1.56	0.12
Handgrip					0.244	2.29	0.023
Gait speed					0.197	2.29	0.023
	Step 3	0.307	8.28	<0.001			
Age					−0.029	−0.37	0.70
Gender ^1^					0.164	1.61	0.10
Education					0.283	2.46	0.015
Handgrip					0.287	2.90	0.004
Gait speed					0.145	1.80	0.073
CRIq					0.418	3.36	0.001
TIB					0.271	3.14	0.002

MMSE = Mini Mental State Examination; BADL = Basic Activities of Daily Life; IADL = Instrumental Activities of Daily Life; FI = Frailty Index; CRIq = Cognitive Reserve Index Questionnaire; TIB = Test di Intelligenza Breve. ^1^ The variable “gender” was dichotomized as “0 = male”, “1 = female”.

## Data Availability

The raw data supporting the conclusions of this article will be made available on request by the corresponding author, without undue reservation.

## Supplementary Materials

The following are available online at https://www.mdpi.com/article/10.3390/brainsci11070824/s1, Table S1: Variables checked in the calculated Frailty Index (FI); Table S2: Pearson’s correlations of cognitive reserve and premorbid IQ indexes at baseline.
