# Supplementary material for: Impact of Cognitive Reserve and Premorbid IQ on Cognitive and Functional Status in Older Outpatients
Source: Brain Sci. 2021 Jun 22;11(7):824. doi: 10.3390/brainsci11070824 (PMC8301973; doi:10.3390/brainsci11070824)
Supplement: Supplementary file 1 [file brainsci-11-00824-s001.zip › brainsci-1259953-supplementary.pdf]

Supplementary files

Impact of Cognitive Reserve and Premorbid IQ on Cognitive and Functional Status in Older Outpatients

Maria C. Quattropani [1], Alberto Sardella [1,*], Francesca Morgante [1,2], Lucia Ricciardi [2,3], Angela Alibrandi [4], Vittorio Lenzo [5], Antonino Catalano [1], Giovanni Squadrito [1] and Giorgio Basile [1]

Table S1. Variables checked in the calculated Frailty Index (FI).

Hospitalization	Pain	Urinary incontinence	Heart failure	Cerebrovascular disease
Fractures	Bathing	Faecal incontinence	Chronic Obstructive Pulmonary Disease	Handgrip strength
Caregiver	Dressing	Telephone	BMI	Parkinsonism
Cognitive status	Walking	Drugs	Cancer	Gait speed
Malnutrition	Getting up / Sitting down	Hypertension	Cirrhosis	Medications
Dehydration	Feeding	Diabetes	Chronic kidney failure	Benzodiazepines
Oral health	Toileting	Heart disease	Obesity	Neuroleptics
			**Total number of detected deficits**	**Frailty Index**
			__ / 35	——

Note*:* The FI is expressed as a ratio of health deficits present to the total number of deficits considered; the greater the number of health deficits, the higher the degree of frailty. According to this approach, patients with a FI ≥0.25 are commonly considered frail.

Table S2. Pearson’s correlations of cognitive reserve and premorbid IQ indexes at baseline.

	CRIq		TIB	
	r	p	r	p

MMSE	0.405	< 0.001	0.387	< 0.001
Handgrip (kg)	0.319	< 0.001	0.099	ns
Gait speed (m/s)	0.260	0.002	0.195	0.02
BADL[1]	0.276	0.001	0.104	ns
IADL[1]	0.329	< 0.001	0.245	0.003
FI[2]	-0.329	< 0.001	-0.225	0.007

Note. MMSE = Mini Mental State Examination; BADL = Basic Activities of Daily Life; IADL = Instrumental Activities of Daily Life; FI = Frailty Index; CRIq = Cognitive Reserve Index Questionnaire; TIB = Test di Intelligenza Breve; m/s= meter per second.

[1]BADL and IADL scores express the number of maintained functions by the subject. [2]Lower FI scores correspond to a better health status.
